# Vaccine sero-monitoring and sero-surveillance of Peste des petits ruminants in small ruminants in West Gojjam zone, Amhara region, Ethiopia

**DOI:** 10.3389/fvets.2024.1392893

**Published:** 2024-12-13

**Authors:** Mesafint Mandefro, Saddam Mohammed Ibrahim, Demeke Sibhatu, Nebiyou Kassa, Kemal Emiyu, Kebede Debebe, Bereket Dessalegn, Mastewal Birhan, Molalegne Bitew

**Affiliations:** ^1^Metema District Livestock and Fishery Resource Development Office, Metema, Ethiopia; ^2^College of Veterinary Medicine and Animal Sciences, University of Gondar, Gondar, Ethiopia; ^3^Serology Laboratory, Animal Health Institute, Sebeta, Ethiopia; ^4^Department of Health Biotechnology, Bio and Emerging Institute Technology, Addis Ababa, Ethiopia

**Keywords:** Amhara National Regional State, PPR eradication, seroconversion, seroprevalence, small ruminants, vaccine monitoring

## Abstract

**Background:**

Peste des petits ruminants (PPR) is an acute or subacute, highly contagious, and economically important, transboundary disease of small ruminants caused by Peste des petits ruminants virus (PPRV).

**Objectives:**

The objective of this study was to determine the seroconversion rate in PPR vaccinated flock of sheep (Sekela district) and the seroprevalence of PPRV in unvaccinated flocks of sheep and goats (Yilmanadensa district).

**Methods:**

A cross-sectional study was conducted from January to March 2022 in two selected districts of West Gojjam zone, Ethiopia. Multistage cluster sampling was used to select sampling units by successively selecting districts, kebeles, and villages purposively based on their accessibility and vaccination status. Individual animals were selected haphazardly mimicking simple random sampling. Accordingly, a total of 660 blood samples were collected. Out of this, 300 sheep were vaccinated 4 months prior to sampling using the Nigerian 75/1 strain-based freeze-dried live attenuated PPR vaccine and 360 small ruminants (288 sheep and 72 goats) were unvaccinated and assayed for anti-PPRV antibodies using commercial c-ELISA.

**Results:**

The post-vaccination herd immunity was 76.66% (95% CI: 71.46–81.34), which is slightly lower than the threshold herd immunity recommended by the PPR global control and eradication strategy, which is set to be 80%, to efficiently break the epidemiological cycle of the virus. Seroprevalence of PPRV in unvaccinated sheep and goats was 3.61% (95% CI: 1.94–6.1), indicating the possible circulation of PPRV in the area. Although small ruminants develop solid immunity following natural infection in endemic countries, the infection of naïve animals allows continuous circulation of the virus.

**Conclusions:**

In light of the accumulating evidence of low post-vaccination herd immunity in small ruminants in Ethiopia, the undergoing PPR vaccination strategy needs to be reevaluated to achieve the desired herd immunity at any time ultimately aiding the eradication goal by 2030.

## 1 Introduction

Peste des petits ruminants (PPR) is an acute or subacute, highly contagious, and economically important transboundary viral disease that inflicts a significant constraint on sheep and goat production in Africa and Asia (1). The disease is caused by Peste des petits ruminants virus, under the genus *Morbillivirus* and family Paramyxoviridae (2). The virus has a close antigenic resemblance to the Rinderpest virus of cattle, the Measles virus of humans, and the Canine Distemper virus of pets and other wild animal species (2). Affected animals exhibit sudden depression, fever, oculo-nasal discharge, stomatitis, respiratory distress, profuse diarrhea, and death (3). The disease could affect up to 100% of animals within the flock, and an outbreak can kill between 20% and 90% of exposed animals (4). Consequently, the disease leads to significant economic, food security, and livelihood impacts in affected communities (5).

In Ethiopia, clinical and serological evidence of PPR has been reported by Taylor (6), and later the disease was confirmed in 1991 in clinical specimens collected from an outbreak around the capital, Addis Ababa (7). Since then, PPR has been spreading through the country with greater impact on small ruminants (8–10).

The devastating socio-economic impact of the disease, coupled with its vast geographical coverage, has called for the progressive control and eradication of PPR at the regional or global level (3, 11, 12). Concordantly, a PPR working group was formed in 2011 by the World Organization for Animal Health (WOAH, the former OIE) and the United Nations Food and Agriculture Organization (FAO) after a discussion on the possibility of PPR progressive control leading to eradication. Accordingly, in March 2015, OIE and FAO officially launched a new program to eradicate PPR by 2030 and presented a global control and eradication strategy (GCES) (13).

Lessons learnt from the eradication of rinderpest were used to set the framework for PPR eradication by 2030. Of note, PPR has several features of an eradicable disease like that of rinderpest. Among these features, PPR is known to be caused by only one viral serotype, and the current PPR vaccines are known to induce protection against all viral lineages and immunity following natural infection or vaccination is lifelong (14); the principal means of transmission of the disease is via direct contact (15); the virus is fragile outside the host and is sensitive to heat and sunlight (16); there is no carrier state and infected animals are infectious for a brief period of time (6); the is no established evidence for the maintenance of the virus in the wildlife and subsequent transmission to domestic small ruminants (17, 18); safe, effective, and thermostable PPR vaccines are available (19, 20); and diagnostic tests of high sensitivity and specificity are available (21). Owing to availability of effective live-attenuated PPR vaccines that induces a solid immunity after a single shot, the WOAH/OIE-FAO global strategy is largely dependent on strategic mass vaccination of small ruminant populations.

As a PPR-endemic country harboring the 7th largest number of small ruminants (https://www.fao.org/faostat/en/#home), Ethiopia, is committed to achieve PPR eradication by 2030 through the implementation of risk-based vaccination of sheep and goats using live-attenuated PPR vaccine following the recommendations of the PPR GCES. However, there are several factors that could challenge the efforts underway, thus periodic evaluation of the ongoing activities is imperative as it will aid, uncovering problems timely, and act accordingly.

The aim of this study was to estimate post-vaccination seroconversion rat in PPR vaccinated flocks of sheep in Sekela district of West Gojjam zone, Amhara region, Ethiopia, and seroprevalence of PPRV in unvaccinated sheep and goats in a neighboring district, Yilmanadensa district.

## 2 Materials and methods

### 2.1 Study area

**Figure 1:**
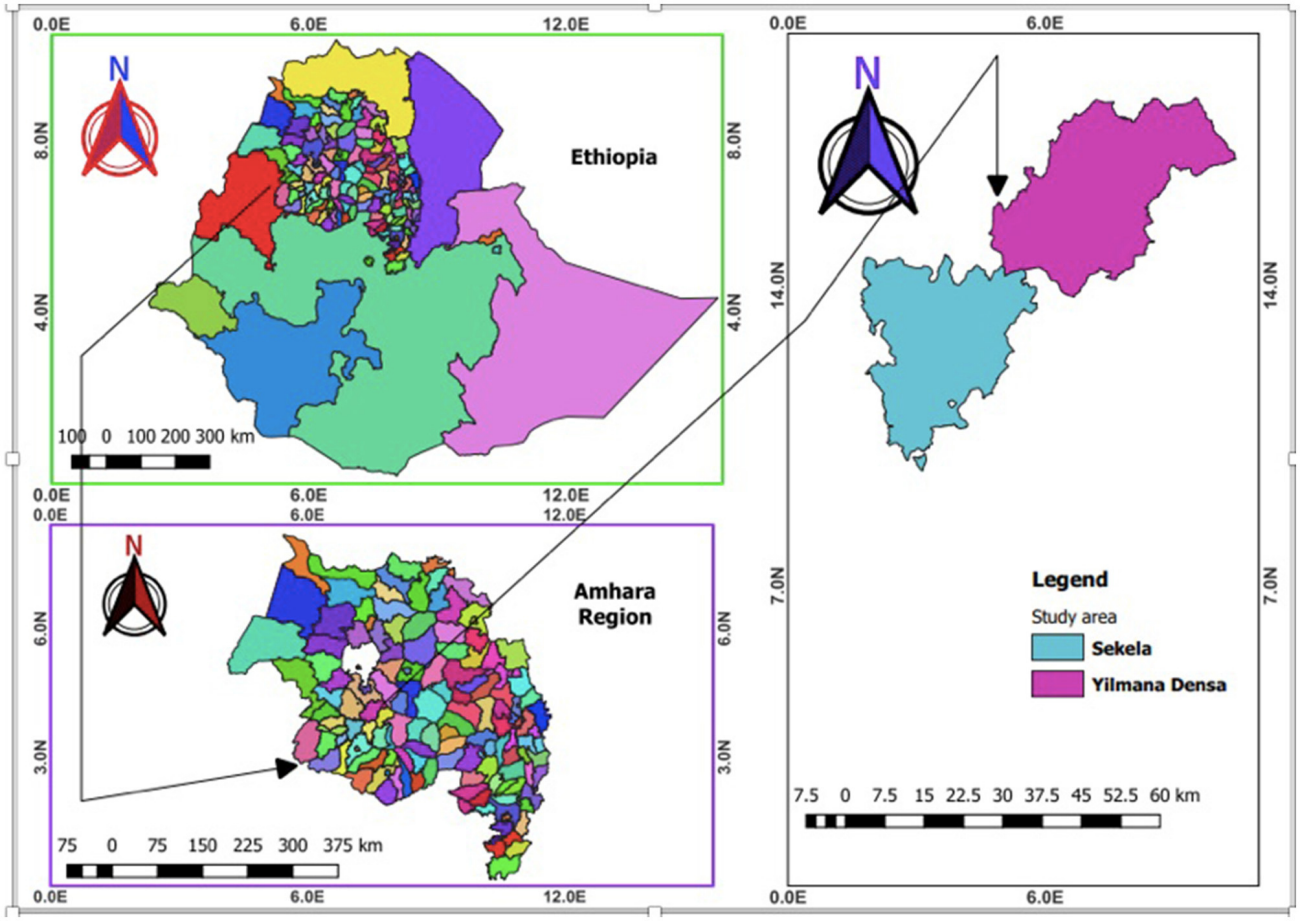
Map of Ethiopia (**upper left**) showing the study districts (**right**) in the Amhara National Regional State (**bottom left**) (created using QGIS version 2.18.1 Las Palmas).

This study was conducted in Sekela and Yilmanadensa districts in West Gojjam zone of the Amhara National Regional State, Ethiopia (Figure 1). In these areas, small ruminants are reared mainly under an extensive management system. Sekela district is located in Northwestern Ethiopia and lies between 10°55′00^′′^ to 11°05′00^′′^ North latitude and 37°55′00^′′^ to 37°31′60^′′^ East longitude. Yilmanadensa district is located 443 km away from Addis Ababa and 43 km Northeast of Bahir Dar city. The district is found between 11°29′59.99^′′^ North latitude and at 37°19′ 60.00^′′^ East longitude.

### 2.2 Study animals

A total of 660 sheep and goats of mixed sex, aged above 6 months of old, and that were managed extensively were involved in this study. For the post-vaccination sero-monitoring study, 300 sheep were sampled from Sekela district, a district that implemented mass vaccination of sheep using the attenuated homologous Nigerian 75/1 PPR vaccine (National Veterinary Institute, Debrezeit, Ethiopia). On the other hand, 288 sheep and 72 goats, all unvaccinated, were sampled to detect PPRV-specific serum antibodies using c-ELISA.

### 2.3 Study design, sampling technique, and sample size estimation

A cross-sectional study design was applied from January to March 2022. Districts that were targets of the PPR vaccination campaign under the PPR GCES framework were selected purposively. Sekela district was selected for the sero-monitoring study because vaccination was already conducted in the majority of the kebeles (administrative divisions under districts). A neighboring and unvaccinated district, Yilmanadensa, was considered for the sero-surveillance study. Within Sekela district, 5 kebeles (including a total of 27 villages) that participated in PPR vaccination campaign were selected purposively based on the convenience of accessibility. Five kebeles (27 villages) within Yilmanadensa district were also selected similarly. Lastly, study animals from the selected kebeles were selected haphazardly to emulate random sampling. Strict random sampling was not possible due to the difficulty in establishing a sampling frame in the traditional extensive production system.

#### 2.3.1 Sample size estimation

Sample size was calculated using the formula provided by Bennett et al. (22) as followed:


$$\begin{array}{l} n=gc=\frac{P\left(1-P\right)D}{SE^{2}} \end{array}$$

Where “*n*” is the sample size, “*p”* is the prevalence as a percentage, “*D”* is the design effect, “*SE*” is the standard error, “*g”* is the average number of individuals sampled per cluster, and “*c”* is the number of clusters which is villages in this case.


$$\begin{array}{l} D=1+\left(g-1\right)ICC \end{array}$$

The estimate of intracluster correlation coefficient (ICC) for most infectious diseases does not exceed 0.2 (23). So, taking 0.2 for the cluster (village) and considering the possibility to collect about 11 serum samples (g) in a village, D equaled 3. We considered an expected sero-prevalence of 50% for PPRV and a vaccine seroconversion rate of 50% (as there are no previous reports in the particular study districts), and a standard error of 0.05 to estimate the number of required clusters. Accordingly, the number of clusters for sero-monitoring and sero-surveillance became 27 with a total number of samples approximating 300 for both objectives. However, the actual number of samples collected for sero-surveillance was increased to 360, due to access and consent of animal owners. Therefore, a total of 660 blood samples were obtained.

### 2.4 Blood sampling

Five (5) ml of blood sample was drawn from the jugular vein of each animal using a sterile needle and a plain vacutainer tube labeled with identification number, species, sex, and age. Blood samples containing tubes were placed tilted at room temperature until the clot was fully separated from the serum. Clear straw-colored serum was decanted into Cryovials, labeled, and placed in an icebox, and transported to Bahir Dar Regional Veterinary Laboratory Center and stored in a deep freezer (−20°C). Then, serum samples were shipped in a cool box chilled with ice packs to the Serology Laboratory of Animal Health Institute (AHI) (Sebeta, Ethiopia) for serological analysis.

### 2.5 Detection of PPRV-specific antibodies

Sera samples were analyzed for the presence of PPRV-specific antibodies (anti-PPRV nucleoprotein antibodies) using a commercial c-ELISA kit (IDvet Screen^®^ PPR competition kit, 310 rue Louis Pasteur, 34790 Grabels, France). The absorbance (optical density) of serum samples was measured using ELISA microplate reader (Multiskan) with an inference filter of 450 nm. According to the manufacturer's estimate, the assay's diagnostic sensitivity and specificity were 94.5 per cent and 99.4 per cent, respectively. Sample positivity or negativity was determined by calculating the sample to negative (S/N) ratio according to the method provided by the manufacturer as follows:


$$\begin{array}{l} \frac{S}{N}=\left(\frac{ODS}{ODNC}\right)*100 \end{array}$$

Where ODS = optical density of the sample, ODNC= optical density of negative control. Samples with S/N ≤ 50% were considered positive, while S/N >60% was negative. S/N values of >50% and ≤ 60% were interpreted as doubtful.

### 2.6 Ethical consideration

This study did not involve any experimental procedures or interventions on live animals. Instead, it utilized blood samples collected by qualified animal practitioners. The collection of these samples did not involve additional harm or distress to the animals. Therefore, as per the guidelines of the Animal Health Institute, the study was deemed exempt from the requirement for formal animal ethical clearance.

### 2.7 Data analysis

Apparent seroprevalence was calculated by dividing the number of seropositive samples by the total number of animals sampled as followed:


$$\begin{array}{l} \text{Apparent Prevalence}=\frac{\text{Number of Seropositive Samples}}{\text{Total Numbers of Animals Sampled}}*100 \end{array}$$

The apparent prevalence (Ap) was adjusted for the sensitivity (Se) (94.5%) and specificity (Sp) (99.4%) of the c-ELISA test to obtain the true prevalence using the formula described by (24).


$$\begin{array}{l} \text{True prevalence}=\;\frac{\text{AP}+\left(\text{SP}-1\right)}{\text{Sp}+\left(\text{Se}-1\right)}*100 \end{array}$$

## 3 Results

### 3.1 Post-vaccine seroconversion

**Figure 2:**
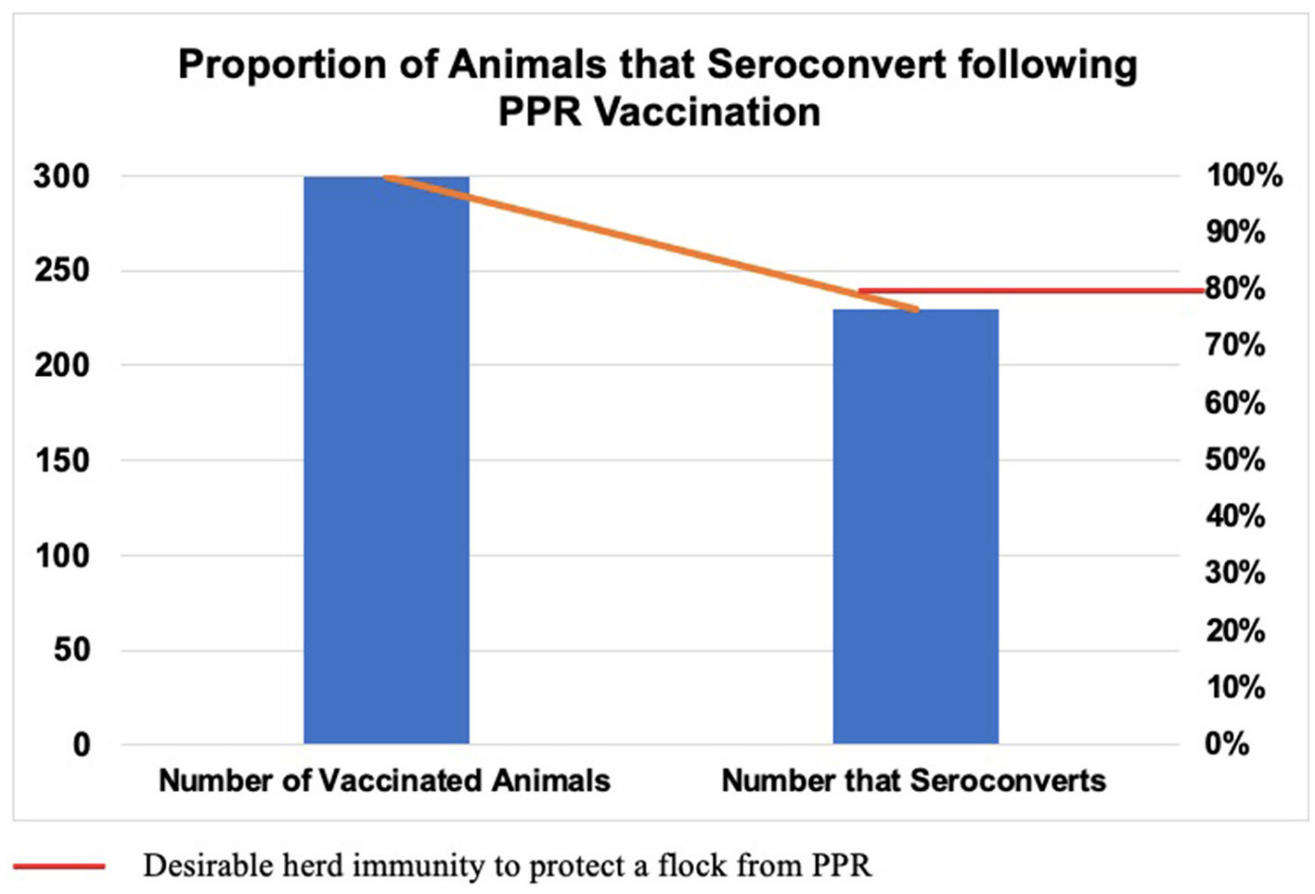
Proportion of animals that seroconvert following vaccination using attenuated homologous PPR vaccine (Nigeria 75/1 strain). Sheep (*n* = 300) were sampled 4 months post-vaccination and serum was analyzed using c-ELISA. Of the total, 230 (76.66%) animals seroconverted, which is slightly lower than the desirable threshold of herd immunity (80%).

In this study, serum was collected from 300 PPR-vaccinated sheep, and the seroconversion rate determined. Sheep were vaccinated with live-attenuated homologous PPR vaccine (Nigeria 75/1 strain) 4 months prior to sampling. Of the 300 sampled sheep, 230 (76.66%) were found to seroconvert in response to vaccination (Figure 2).

### 3.2 Seroconversion rate by kebele divisions

Analysis of vaccine seroconversion rate by kebele divisions showed higher rate in kebeles, Gundil and Sawsa (91.67%) followed by kebeles Abisken (76.67%), Ambicy (68.33%), and Kolelecha (55%). The differences across kebeles were statistically significant (*p* < 0.05) (Table 1).

**Table 1:** PPR vaccine seroconversion rate in sheep flock in different kebeles of Sekela district of West Gojjam Zone.

**Kebeles**	**Number of tested samples**	**Numbers that seroconverted**	**Percent proportion (95% CI)**	**True prevalence (%)**
Kolelecha	60	33	55 (41.6–67.88)	
Ambicy	60	41	68.33 (55.04–79.74)	
Gundil	60	55	91.67 (81.61–97.34)	
Abisken	60	46	76.67 (63.96–86.61)	
Sawsa	60	55	91.67 (81.61–97.34)	
Total	300	230	76.66 (71.46–81.34)	

Kebele: lowest administrative division; X^2^ = 33.16 (df = 4, p = 0.001).

### 3.3 Seroprevalence of PPRV in unvaccinated flocks

In addition to measuring the vaccine response of flocks, this study collected and analyzed 360 sera from unvaccinated sheep (*n* = 288) and goats (*n* = 72), to estimate the seroprevalence of PPRV in a neighboring district close to the vaccinated district. The overall apparent prevalence of PPRV was 3.61% (13/360). After adjusting for the PPR c-ELISA specificity and sensitivity, the true overall prevalence became 3.2%. The species level apparent prevalence of PPRV antibodies in sheep and goats was also 3.47% (10/288) and 4.16% (3/72), respectively. The true species level prevalence was 3% and 3.8% in sheep and goats, respectively.

### 3.4 Seroprevalence of PPRV by kebele divisions

Antibodies to PPRV were detected in unvaccinated small ruminants from all kebeles sampled. The highest sero-prevalence was recorded in Koti kebele (6.94%) followed by Senkegna (5.55%), Walka and Agta kebele (2.77%), and Abka (1.4%). However, this difference in seropositivity across kebeles was statistically insignificant (*p* > 0.05) (Table 2).

**Table 2:** Seroprevalence of PPRV in unvaccinated sheep and goats in Yilamanadensa district of West Gojjam zone.

**Kebeles**	**Samples tested**	**Positive sample**	**Apparent prevalence% (95% CI)**	**True prevalence%**
Senkegna	72	3	4.2 (0.87–11.7)	3.8
Walka	72	2	2.8 (0.34–9.7)	2.3
Koti	72	5	6.9 (2.29–15.5)	6.7
Abka	72	1	1.4 (0.04–7.5)	0.9
Agta	72	2	2.8 (0.34–9.7)	2.3
Total	360	13	3.6 (1.94–6.1)	3.2

Kebele: lowest administrative division; X^2^ = 3.6710 (df = 4, p = 0.452).

## 4 Discussions

PPR is a disease of high socio-economic importance that has a global impact (25). Several regional and global efforts are underway to control and ultimately eradicate the disease by 2030 (3, 11, 12, 14). Among the control and prevention strategies, vaccination is the most feasible and effective option to control PPR in resource-limited countries such as Ethiopia. Direct contact being the main route of transmission (15), movement control is also effective but is difficult to implement in many of the affected countries, including Ethiopia, where extensive and mobile production systems are common.

Recently, a global initiative has been put in place to eradicate PPR by 2030, using vaccination as the main tool. Being a potential hub for small ruminant production and having an enormous population of small ruminants, the Amhara National Regional State is among the targets of the PPR eradication program in Ethiopia. The PPR global strategy recommends sero-monitoring as one tool of post-vaccination evaluation (PVE). It necessitates the assessment and monitoring of population immunity regularly, depending on the country's epidemiological situation, budget, and needs (13).

In line with this, a study to evaluate the post-vaccination herd immunity of small ruminants against PPR was initiated in the Metema district in the Northwest Amhara region (26). This sero-monitoring study was conducted in PPR-vaccinated flocks of sheep in the West Gojjam zone of the Amhara region, a previously unaddressed area. Thus, we believe the findings of this study could stimulate further research that helps evaluate the ongoing efforts or strategies to control and eradicate PPR in the country.

In this study, the seroconversion rate in response to the PPRV Nigerian 75/1 vaccine strain was 76.66% (95% CI: 71.46–81.34). Additionally, seroconversion rates varied across different kebele divisions significantly (*p* < 0.05). A higher seroconversion rate was recorded in Gundil and Sawsa (91.67%), followed by Abisken (76.67%), Ambicy (68.33%), and Kolelecha (55%). Even if the first two kebeles seemed to achieve higher post-vaccine seroconversion (91.67%), this is not expected to provide protection in the field because kebeles are not epidemiological units but rather administrative divisions. Practically, animals from different kebeles tend to share grazing and watering points; thus, in this study, all five kebeles are collectively considered as a single epidemiological unit. Consequently, the overall post-vaccine seroconversion (76.66%) would have more practical significance. This finding is slightly lower than the desired level of herd immunity recommended by the PPR GCES, which entails that 80% of the vaccinated herds should seroconvert to result in an efficient protection barrier (13).

The Nigerian 75/1 strain-based PPR vaccine is an effective vaccine and has successfully been used in the control of PPR in several African countries (27–29). However, different factors could contribute to lower-than-expected seroconversion rate in a population of immunized animals at any given time. In this case, as the vaccine is live, it is vulnerable to death or inactivation due to cold-chain failure, an issue quite common in resource-limited countries (30). In addition, herd immunity could be affected due to the rapid turnover seen in small ruminants (26). The introduction of new animals into a herd through purchase or gift is a common practice in Ethiopia. Thus, unvaccinated animals entering the vaccinated herd could lower the population immunity. Similarly, the withdrawal of vaccinated animals for the same reasons could dilute an already-formed herd immunity. Furthermore, some animals could fail to mount an effective response to vaccination due to their genetic make-up (31) or underlying morbidities impairing immunity (32), consequently affecting the desired seroconversion rate and protective barrier at the herd level.

In accordance with the present study, Luka et al. (33) reported an insufficient sero-conversion rate of 55.26% among PPR-vaccinated small ruminants in Uganda. In addition, the finding of this study is consistent with two other studies in Ethiopia: Yirga et al. (26) and Faris et al. (34), who reported a lower herd immunity than the PPR GCES's threshold herd immunity in vaccinated small ruminant flocks in Metema district, Amhara region (68%) and Awash Fentale district, Afar region (61.13%), respectively. Of note, the mathematical transmission model of the rinderpest virus has shown that suboptimal vaccination coverage is likely to favor virus persistence (25). Thus, the suboptimal herd immunity reported in this and other studies (26, 34) calls for an urgent evaluation of the ongoing strategy to control and ultimately eradicate PPR in Ethiopia.

In agreement with our report, Balamurugan et al. (35) documented a seroconversion rate of 73.4% in vaccinated small ruminants in India. In Somalia, another large study evaluated the effectiveness of PPR vaccination in small ruminants, revealing an increase in seroprevalence from 62 to 72% after mass immunization (36). On the other hand, in the central and western regions of India, Balamurugan et al. (37) reported a seroconversion rate ranging from 21 to 74%, which could be due to differences in vaccination frequency and pattern. Ahamed et al. (38) reported an increase in vaccination-induced seropositivity in goats from 48% to 96% 21 days post-vaccination. However, this was an experimental study conducted on a small group of animals; therefore, it would not be reasonable to compare it with field studies.

Another key component of the PPR eradication program is an appropriate disease surveillance system to detect virus incursion or virus circulation, particularly in unvaccinated parts of the national population, as it will aid in adequately interpreting the PVE results. In the present study, an overall seroprevalence of 3.61% (13/360) was recorded in unvaccinated sheep and goats in Yilmanadensa district, indicating present or past exposure of animals to PPRV in the area. Even though small ruminants develop solid immunity following natural infection in endemic countries, the infection of naïve animals (e.g., young-of-the-year) allows continuous circulation of PPRV (39).

This finding (3.61%) is comparable with the reports of Fentie et al. (40) in Mecha district (3.70%) of West Gojjam zone, Gebre et al. (41) in Bench Maji and Kata zones (2.1%), and Faris et al. (34) in Awash Fentale district in Afar region, Ethiopia. Contrarily, higher seroprevalences were recorded in the Eastern Showa and Arsi zones, the Oromia region, Ethiopia (48.43%) (42), the South Omo zone, Sothern Ethiopia (48.43%) (43), and selected districts of the Afar region, Ethiopia (60.15%) (44). This variation can be explained by sample size differences, geographical and seasonal effects, host population density, disease control programs, and the social environment that can influence contact rates and husbandry practices.

As a limitation, the baseline serum status of vaccinated sheep was not documented due to the rapid turnover of small ruminants in the area, making it difficult to keep a record and resample after a defined period of time. Therefore, the impact of a probable prior infection with PPRV on the vaccine response of individual animals was not estimated. However, from the sero-surveillance study of this and other studies in the region (mentioned above), a baseline prevalence of more than 5% is not expected.

## 5 Conclusions

This study reported a suboptimal post-vaccine seroconversion (76.66%) in PPR-vaccinated flocks of sheep in Sekela district of West Gojjam zone. In light of the accumulating evidence of lowered herd immunity in vaccinated small ruminants in Ethiopia, the PPR vaccination strategy needs to be reevaluated to achieve the threshold herd immunity set by the PPR global eradication program, thus breaking the epidemiological cycle of the virus. The vaccine cold chain and other technical aspects of the vaccination strategy should be assessed for prompt intervention. As the population dynamics of small ruminants are higher and characterized by rapid turnover of flocks, increasing the frequency of vaccination that involves immunization of newcomers could help sustain a protective herd immunity within a flock. Estimation of seroprevalence alongside the vaccination campaign is also another key requirement to augment the objectives of the PPR GCES. An overall seroprevalence of 3.6% was reported in this study, reflecting the possible circulation of PPRV and thus demanding an appropriate intervention. Future studies are encouraged to explore and identify key operational elements of vaccination campaigns that will help increase the success rate.

## Data Availability

The original contributions presented in the study are included in the article/supplementary material, further inquiries can be directed to the corresponding author.
